# Azole Antifungal Consumption in Community Pharmacy Sales in Mainland Portugal: Trend Analysis from 2014 to 2023

**DOI:** 10.3390/antibiotics14010033

**Published:** 2025-01-04

**Authors:** Sofia Moura, Paulo Duarte, Ana Sofia Oliveira, José Martinez-de-Oliveira, Ana Palmeira-de-Oliveira, Joana Rolo

**Affiliations:** 1 Faculty of Health Sciences, University of Beira Interior, 6200-506 Covilhã, Portugal; sofia.moura@ubi.pt (S.M.); ana_g2s@hotmail.com (A.S.O.); apo@fcsaude.ubi.pt (A.P.-d.-O.); 2 CICS-UBI—Health Sciences Research Centre, University of Beira Interior, 6200-506 Covilhã, Portugal; jmo@fcsaude.ubi.pt; 3 Department of Clinical Pathology, Santarém District Hospital, Lezíria Local Healthcare Unit, 2005-177 Santarém, Portugal; 4 RISE-Health, Faculdade das Ciências da Saúde, Universidade da Beira Interior, 6200-506 Covilhã, Portugal; 5 NECE—Research Centre for Business Sciences, Faculty of Human and Social Sciences, University of Beira Interior, 6200-209 Covilhã, Portugal; pduarte@ubi.pt; 6 Labfit, Health Products Research and Development Lda, 6200-284 Covilhã, Portugal

**Keywords:** fluconazole, isoconazole, itraconozole, Portugal, sertaconazole

## Abstract

**Background/Objectives**: Excessive or inadequate use of antimicrobial drugs may lead to the emergence of resistant strains. For this reason, it is important to monitor consumption indicators to assess drugs’ utilization over time. This study aimed to analyze the consumption of medically prescribed azole antifungal drugs in mainland Portugal from 2014 to 2023, focusing on those directed to genital infections: fluconazole, isoconazole, itraconazole, and sertaconazole. **Methods**: For each drug, the evaluated parameters were the total number of packages, number of packages per 1000 inhabitants, defined daily dose (DDD) per 1000 inhabitants per day, and total costs. For this purpose, we used data from community pharmacies’ sales, which are available through INFARMED (the Portuguese national authority on medicines and health products). **Results**: Several trends emerged from data analysis. The COVID-19 pandemic negatively affected the consumption of all azole antifungal drugs included in this study. However, after 2020, fluconazole and sertaconazole consumption has been increasing. In the specific case of fluconazole, there was an increase in expenditure, although the total number of packages suffered a decrease over the 10-year study period. Additionally, the defined daily dose (DDD) per 1000 inhabitants per day for fluconazole and itraconazole was lower compared to estimates from the last available survey (2009). **Conclusions**: Although our findings represent a lesser pressure on fungi, further monitoring is needed to better understand the evolution of fluconazole and itraconazole consumption over time, particularly due to the trends observed in this study.

## 1. Introduction

Fungal infections are a growing public health threat, yet they receive far less attention than bacterial and viral diseases and depend on fewer resources worldwide. One of the mechanisms to fight these types of infections is the use of antifungal drugs. Although essential in the medical armamentarium, antifungals must be used rationally to avoid the emergence of resistant strains [1].

Azole antifungal drugs are utilized to fight different types of fungi, such as yeast infections, like Vulvovaginal Candidosis and Cryptococcosis, and mold infections, like Aspergillosis [2]. Azoles are a class of antifungal agents that inhibit lanosterol 14 alpha-demethylase, which is crucial for the biosynthesis of ergosterol, an essential component of fungal cell membranes. Cell membrane disruption affects its permeability, leading to cell lysis and death [3].

Resistance to azole antifungal drugs can vary significantly depending on the species of fungus, geographic origin, and the pharmacological profile of the specific azole in use. For example, *Aspergillus* spp. and *Candida krusei* isolates are intrinsically resistant to fluconazole. At the same time, resistance rates can differ from region to region in part due to the amounts of azoles present in the environment, for example, in agricultural practices [4].

Five azole antifungal drugs (clotrimazole, fluconazole, itraconazole, miconazole, and voriconazole) are listed in the World Health Organization Model List of Essential Medicines [5]. Their availability and cost vary widely across countries. While fluconazole is widely available, others are less readily available. In 2018, itraconazole and fluconazole were among the systemic antifungal drugs more commonly used in middle- and high-income countries [6].

Within Europe, Portugal was the third country with the highest consumption of outpatient systemic antimycotic and antifungal use in 2009, presenting 0.43 and 0.32 defined daily dose (DDD) per 1000 inhabitants per day for itraconazole and fluconazole, respectively [7]. Azevedo et al. [8] conducted a trend analysis regarding azole antifungal drug consumption in Portugal from 2003 to 2013 and concluded that azole sales under medical prescription were very high. In 2013, fluconazole and itraconazole were the most consumed among azoles subject to medical prescription sold in community pharmacies [8].

With a special focus on azole antifungal drugs used to treat Vulvovaginal Candidosis, the goal of this study is to conduct a trend analysis of fluconazole, isoconazole, itraconazole, and sertaconazole community pharmacies’ sales in mainland Portugal from 2014 to 2023. Understanding antifungal drugs’ consumption patterns is important to assess and interpret the future emergence of antifungal resistance.

## 2. Results

### 2.1. Number of Packages Sold

From 2014 to 2023, fluconazole presented the highest number of packages sold in mainland Portugal, followed by sertaconazole, itraconazole, and isoconazole (Table 1). During this period, the most prescribed fluconazole package was the one containing two capsules of 150 mg each, and for itraconazole, it was the one with 32 capsules of 100 mg each. Regarding sertaconazole, the cream was the most prescribed pharmaceutical form. As for isoconazole vaginal cream, there was a decrease in sales by 98.48% during the same period.

**Figure 1:**
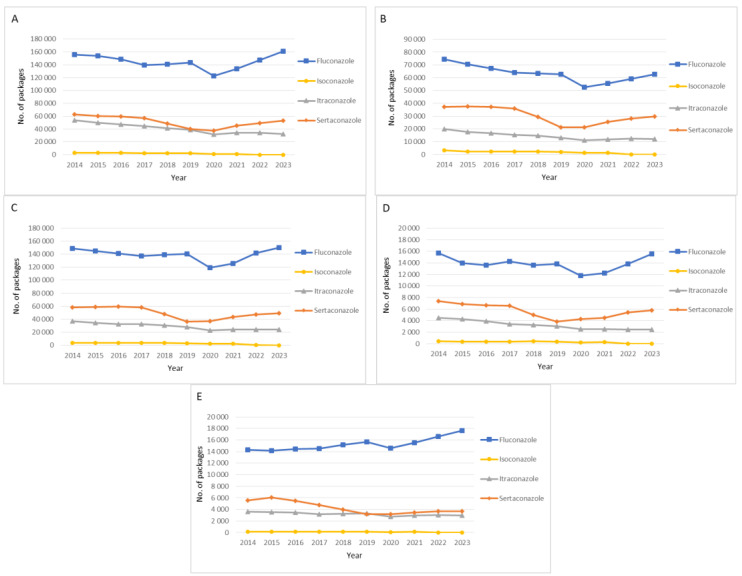
Consumption of fluconazole, isoconazole, itraconazole, and sertaconazole by number of packages and by Regional Health Administration (**A**—North, **B**—Centre, **C**—Lisbon and Tagus Valley, **D**—Alentejo, and **E**—Algarve) between 2014 and 2023.

Figure 1 shows the total number of packages sold in each Portuguese Regional Health Administration (North, Centre, Lisbon and Tagus Valley, Alentejo, and Algarve). Overall, these regions presented similar consumption patterns, such as the most sold azole antifungal drug being fluconazole, followed by sertaconazole, itraconazole and finally isoconazole. Additionally, all regions showed a decrease in fluconazole, isoconazole, and itraconazole consumption in 2020. The exception was sertaconazole that only presented a decrease in the regions North and Algarve.

**Figure 2:**
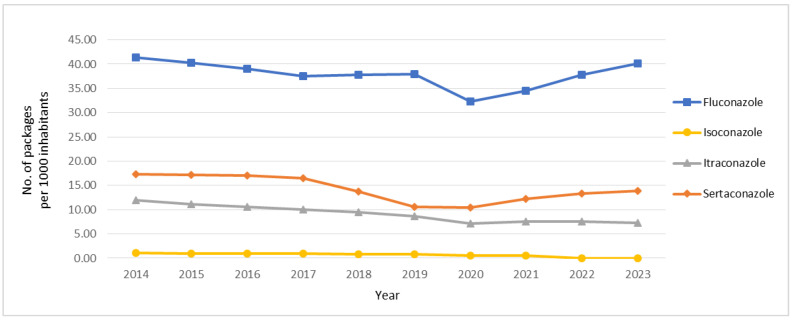
Consumption of fluconazole, isoconazole, itraconazole, and sertaconazole by number of packages per 1000 inhabitants in community pharmacies in mainland Portugal between 2014 and 2023.

As shown in Figure 2, the overall trend for fluconazole shows an accentuated decrease (by 14.79%) in consumption in 2020 compared to the previous year, with a rapid increase in the following years. Comparing the number of packages sold between 2014 and 2023, the difference was −1.23 per 1000 inhabitants. A decrease in sales between 2019 and 2020 was also observed for isoconazole, itraconazole, and sertaconazole, which were 36.25%, 17.43%, and 1.04%, respectively. All azole antifungal drugs included in this study were less consumed in 2023 compared to 2014. Nonetheless, after 2020, sertaconazole and especially fluconazole were on a rising path.

### 2.2. Defined Daily Dose (DDD) per 1000 Inhabitants per Day

Table 2 provides the estimated DDD per 1000 inhabitants per day between 2014 and 2023. Until 2020, the azole antifungal drug with the highest DDD per 1000 inhabitants per day was itraconazole, ranging from 0.380 to 0.235 in 2014 and 2020, respectively. Since 2021, we have observed a shift with fluconazole, which presents higher values for this indicator. For fluconazole, the lowest estimate was 0.233 in 2020, and the highest was 0.292 in 2023. Since 2020, there has been a steady increase of approximately 0.02 for this indicator.

### 2.3. Total Costs

**Figure 3:**
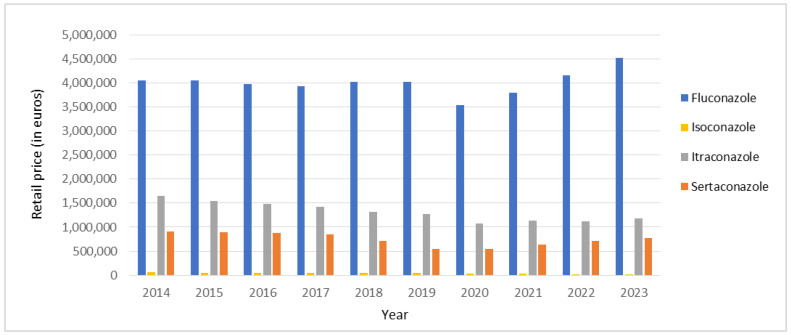
Total costs of fluconazole, isoconazole, itraconazole, and sertaconazole from community pharmacies’ sales in mainland Portugal between 2014 and 2023.

During the 10-year period of this study, the highest expenditure observed was with fluconazole, followed by itraconazole, sertaconazole, and isoconazole (Figure 3). Costs with fluconazole varied from € 3,529,335.79 in 2020 to € 4,523,052.76 in 2023. Fluconazole expenditure in 2023 represented an increase of 11.68% when compared to 2014.

## 3. Discussion

When analyzing the data, our attention was particularly drawn to the decrease in community consumption of all studied azole antifungal drugs during 2020. This decrease may be a consequence of the COVID-19 pandemic. During this year, some non-COVID-19 clinical activity was suspended or postponed due to the pressure on healthcare institutions to respond to increased respiratory symptoms [9]. This led to fewer medical appointments in many medical specialties [10], from which one may assume fewer prescriptions of drugs. On the other hand, in the absence of medical appointments’ availability, patients may have looked for antifungal drugs that were not subject to medical prescription, such as clotrimazole, econazole, fenticonazole, and nistatin. Another possible explanation for this decrease in consumption may be a lower prevalence of infections due to reduced opportunities for human contact due to the implementation of non-pharmaceutical interventions, such as lockdowns where gatherings and meetings were banned and public spaces were closed. This reduction in infection rates could have led to an overall decrease in consumption of broad-spectrum antibiotics, which is a main risk factor associated with recurrent Vulvovaginal Candidosis [11].

Additionally, infection control measures were promoted, such as frequent hand washing, reduction of facial touch, and physical distancing [12]. Due to these restriction measures, fewer fungal infections may have occurred, which led to a decrease in the need to use azole antifungal drugs. These findings highlight how public health emergencies can disrupt standard prescribing patterns, affecting overall antifungal drug usage trends. Future studies should explore the impact of similar disruptions on other drug classes. Health systems can use these insights to adapt prescribing practices and ensure essential services during crises.

After 2020, some azole antifungal drugs included in this study started an ascending trend in consumption, such as fluconazole and sertaconazole. Regarding fluconazole, the number of packages per 1000 inhabitants sold in 2023 was lower compared to 2014; however, the consumption of certain package sizes, such as the 150 mg capsule, increased. For example, the fluconazole pack with two capsules of 150 mg each presented a decrease in consumption, but the one with one capsule of 150 mg increased from 37,292 packages in 2014 to 51,493 in 2023. One may feel tempted to conclude that this could point to a shift in less severe fungal infections, where one capsule is often sufficient to fight the infection, or a change in the type of fungal infection, for example, Vulvovaginal Candidosis can be treated with one capsule of 150 mg. In contrast, other fungal infections could take weeks of treatment. This study does not provide sufficient data to draw a conclusion, as multiple packages may be used for a single fungal infection.

Another trend noticed was the accentuated decrease in isoconazole vaginal cream consumption by 98.48% from 2014 to 2023. We wonder if this reflects a medical preference or if there is another reason for this decrease. The International Society for the Study of Vulvovaginal Disease indicates isoconazole vaginal suppository as one of the available treatment options for patients with uncomplicated Vulvovaginal Candidosis (mild symptoms); however, it does not mention vaginal cream [11].

This study estimates that fluconazole and itraconazole DDD per 1000 inhabitants per day are lower than 2009 levels, as reported by Adriaenssens et al. [7]. In 2009, fluconazole and itraconazole DDD per 1000 inhabitants per day were 0.32 and 0.43, respectively. Our findings show a lower DDD per 1000 inhabitants per day for both drugs in 2023. This decrease could represent a lesser antimicrobial pressure on fungi, which is a good sign for the maintenance of antifungal effectiveness, as excessive use could lead to the emergence of resistant strains. As seen in other indicators, after 2020, there was also an increase in DDD per 1000 inhabitants per day for fluconazole and itraconazole. However, the evolution of this indicator in these two drugs was different. Fluconazole presented a rapid increase, surpassing the estimate at the beginning of this study period, 0.292 in 2023 compared to 0.271 in 2014. As for itraconazole, there was a modest increase; however, the highest value was estimated to be in 2014. Further monitoring of these indicators is needed to better understand the evolution of these drugs’ consumption over the next years. Comparing our indicators with global consumption of antifungal agents in 65 middle- and high-income countries [6], Portugal presented a higher fluconazole DDD per 1000 inhabitants per day. In 2018, the global fluconazole consumption was 0.2296 DDD per 1000 inhabitants per day, while in Portugal, it was 0.265. Regarding itraconazole, Pathadka et al. [6] showed an increase in DDD per 1000 inhabitants per day during their study period, which is contrary to the trend shown in our study. In 2018, the global consumption of itraconazole was 0.324 DDD per 1000 inhabitants per day, while in Portugal, it was 0.297.

Monitoring consumption trends is important to understand the emergence of resistance patterns. In Portugal, several studies report resistance to azole antifungal drugs [13,14,15,16,17,18,19,20,21,22,23,24,25,26,27,28], which need to be carefully interpreted in terms of species, type of samples, provenience, and time period. The most recent ones showed different percentages of resistance to fluconazole and itraconazole. A study published by Azevedo et al. [13] found that 3.9% of yeasts isolated from the oral cavity, gut, and breastmilk were resistant to fluconazole. Rolo et al. [14] showed that 46% of vaginal *Candida* spp. isolates were resistant to fluconazole. Simões-Silva et al. [15] reported that all oral *Candida* isolates identified in their study were susceptible to fluconazole and resistant to itraconazole. Sabino et al. [16] conducted a trend analysis and found that, between 2012 and 2019, 4.4% of *Aspergillus fumigatus* sensu stricto isolates were resistant to itraconazole. Focusing only on Vulvovaginal Candidosis isolates, considering the same region in Portugal, two consecutive studies have revealed that *C. albicans* isolates collected between 2009 and 2014 had a fluconazole resistance rate of 61% [14], while isolates collected between 2019 and 2022 had a fluconazole resistance rate of 8.9% [17]. On the other hand, at the same time period, the fluconazole resistance rate among *C. glabrata* isolates increased from 3% in the first study to 53% in the second study. It would be interesting to verify if this trend is statistically significant and related to our findings in the current study. Further research is needed to consolidate these findings.

As for costs related to azole antifungal drugs included in the current study, fluconazole expenditure increased by 11.68% between 2014 and 2023. Although there was a decrease in the total number of packages sold, this increase can be explained by the fact that several types of packages presented a rise in sales. For example, the fluconazole pack size containing one capsule of 150 mg increased sales by 38.08% and suffered a growth of 5.36% per package during the 10-year study period. Given that Portugal has a higher fluconazole consumption rate than other countries, future research should investigate the reasons behind this trend and assess whether these patterns correlate with unique epidemiological factors or prescribing behaviors within Portugal. Furthermore, it highlights the importance of healthcare policymakers considering pricing, accessibility, and the economic burden of antifungal medications on public health budgets. Another perspective on this finding would be the need to implement additional measures to prevent a possible rise in fluconazole resistance like, for instance, expanding the availability of generic drugs in the community, reducing inappropriate consumption by raising public awareness with campaigns on the prudent use of antifungals, investment in diagnostics and surveillance to track resistance trends, the inclusion of major fungal diseases in the Portuguese list of notifiable diseases or cross-sectional collaboration between healthcare, agriculture, and policymakers in Portugal.

The main limitations of this study are the following. First, the data used in this study includes only sales in community pharmacies and only for medically prescribed drugs. It would be interesting to analyze data on over-the-counter azole antifungal drugs and observe if their consumption patterns were alike. On the other hand, the dataset did not include the consumption of medically prescribed azole antifungal drugs in hospitals. Since studies report increased fungal infections during the COVID-19 pandemic in hospitals [29,30,31], the decrease in azole antifungal drugs in 2020 should be interpreted cautiously. Second, the Portuguese Law on Epidemiological Surveillance of Communicable Diseases [32] does not include fungal infections. Although there are local studies on the prevalence of these types of infections and their resistance patterns [13,14,15,16,17,18,19,20,21,22,23,24,25,26,27,28], as we mentioned previously in this discussion, there are no data at a national level. For this reason, we could not correlate the indicators used in this study with prevalence and azole resistance patterns over the 10-year study period. Nonetheless, this study shows the trends of azole antifungal drugs community pharmacies’ sales over 10 years, which is important to understand consumption patterns. These data are crucial for determining the need for antifungal stewardship to address the emergence of resistance.

## 4. Materials and Methods

### 4.1. Study Design

This study is a trend analysis of fluconazole, isoconazole, itraconazole, and sertaconazole community pharmacies’ sales in mainland Portugal from 2014 to 2023. These antifungal drugs were selected for inclusion since these are the most used drugs to treat genital infections caused by fungi [13].

### 4.2. Data Source

The analysis is based on the data provided by INFARMED—National Authority of Medicines and Health Products, I.P., a Portuguese government agency. The dataset includes only sales of medically prescribed drugs subject to government reimbursement in community pharmacies, which means that the administration of this type of drug in hospital healthcare was not included. The dataset was limited to drugs sold in mainland Portugal, excluding the Azores and Madeira islands.

The estimated number of inhabitants in Portugal per year was obtained from Statistics Portugal [33].

### 4.3. Data Analysis

Our focus was on azole antifungal drugs used to treat Vulvovaginal Candidosis. Therefore, only fluconazole (capsule and powder for oral suspension), isoconazole (vaginal cream), itraconazole (capsule and oral solution), and sertaconazole (cream, cutaneous powder, cutaneous solution, and pessary) were included in this study. For each of these drugs, the evaluated parameters were the total number of packages, number of packages per 1000 inhabitants, DDD per 1000 inhabitants per day, and total costs.

We conducted a descriptive analysis of the total number of packages sold by pharmaceutical form, strength, pack size, and year for each azole antifungal drug. Additionally, the total number of packages sold was presented by each Regional Health Administration (that includes each geographic region in mainland Portugal). Since we observed the same trends across geographic regions, we focused on estimating DDD per 1000 inhabitants per day for the whole Portuguese population. We estimated the number of packages per 1000 inhabitants for each antifungal drug, dividing the total number of packages sold by the population size, and multiplying by 1000. This indicator was calculated for each year of the study period. DDD was estimated as defined by the World Health Organization Collaborating Centre for Drug Statistics Methodology [34]. To calculate the indicator DDD per 1000 inhabitants per day, the annual number of DDD was divided by the population size and the number of days. Finally, the result was multiplied by 1000. DDD per 1000 inhabitants per day was estimated for fluconazole, isoconazole, and itraconazole. Sertaconazole was excluded from this particular evaluation since there is no available DDD for this drug. Finally, we computed the retail price, in euros, for each azole antifungal drug by year.

## 5. Conclusions

Continued surveillance of antifungal usage trends in Portugal is critical, as is education among healthcare providers regarding conservative prescribing practices. The study’s findings support the need for ongoing monitoring of antifungal usage in Portugal to detect shifts in prescribing practices and guide antifungal stewardship initiatives. Additional research on patient adherence, alternative therapies, and resistance patterns would provide valuable insights for clinical guidelines and public health strategies.

## Figures and Tables

**Table 1:** Number of packages sold in community pharmacies in mainland Portugal for fluconazole, isoconazole, itraconazole, and sertaconazole between 2014 and 2023.

			Year										
Active Substance/ Pharmaceutical Form	Strength	Pack Size	2014	2015	2016	2017	2018	2019	2020	2021	2022	2023	TOTAL
**Fluconazole**													
Capsule	50 mg	7	34,915	34,500	32,747	30,983	31,459	31,984	28,677	30,579	32,539	34,636	323,019
Capsule	100 mg	14	13,618	13,855	14,034	14,660	15,757	16,176	14,859	15,852	16,904	17,408	153,123
Capsule	150 mg	1	37,292	40,071	40,784	35,107	41,777	51,429	35,750	40,026	46,305	51,493	420,034
Capsule	150 mg	2	287,648	273,161	260,727	252,821	246,975	238,558	208,458	220,523	243,195	260,189	2,492,255
Capsule	200 mg	7	14,391	15,314	15,599	14,795	15,753	15,867	13,663	14,960	16,374	17,773	154,489
Capsule	200 mg	14	17,449	16,683	17,406	18,158	18,181	18,748	17,054	18,722	21,155	23,580	187,136
Powder for oral suspension	10 mg/mL	35 mL	1780	1548	1474	902	4	0	0	0	0	0	5708
Powder for oral suspension	40 mg/mL	35 mL	2084	2343	1943	2486	2788	2846	2165	2014	2175	1937	22,781
		TOTAL	409,177	397,475	384,714	369,912	372,694	375,608	320,626	342,676	378,647	407,016	3,758,545
**Isoconazole**													
Vaginal cream	10 mg/g	40 g	11,139	8946	9349	8854	8716	7869	5020	4995	319	169	65,376
**Itraconazole**													
Capsule	100 mg	4	15,615	14,627	13,208	11,981	11,558	8721	4794	4826	5097	5272	95,699
Capsule	100 mg	15	13,217	13,020	11,944	11,625	11,711	10,456	9286	9663	9353	9541	109,816
Capsule	100 mg	16	13,713	12,430	12,382	11,688	11,769	13,872	11,975	13,302	13,654	9917	124,702
Capsule	100 mg	28	35,677	34,172	31,872	31,004	27,597	20,390	18,250	19,028	18,996	13,551	250,537
Capsule	100 mg	32	38,665	33,886	33,133	31,271	29,024	31,397	25,729	27,369	27,919	34,258	312,651
Oral solution	10 mg/mL	150 mL	1705	1525	1456	1512	1326	1393	1251	1374	1171	1578	14,291
		TOTAL	118,592	109,660	103,995	99,081	92,985	86,229	71,285	75,562	76,190	74,117	907,696
**Sertaconazole**													
Cream	20 mg/g	30 g	97,631	97,132	96,599	93,051	68,587	46,536	54,002	66,553	75,387	80,510	775,988
Cutaneous powder	20 mg/g	30 g	19,251	20,049	21,502	21,994	18,964	19,542	16,503	20,048	23,424	25,617	206,894
Cutaneous solution	20 mg/mL	30 mL	18,006	17,080	16,638	16,626	19,898	17,487	14,069	14,443	14,181	14,687	163,115
Pessary	300 mg	1	36,193	35,758	33,533	30,505	27,967	20,988	19,006	20,633	20,848	20,356	265,787
		TOTAL	171,081	170,019	168,272	162,176	135,416	104,553	103,580	121,677	133,840	141,170	1,411,784

**Table 2:** Fluconazole, isoconazole, and itraconazole DDD per 1000 inhabitants per day between 2014 and 2023.

Year	Fluconazole	Isoconazole	Itraconazole
2014	0.271	0.002	0.380
2015	0.266	0.002	0.349
2016	0.262	0.002	0.333
2017	0.261	0.002	0.320
2018	0.265	0.002	0.297
2019	0.266	0.001	0.279
2020	0.233	0.001	0.235
2021	0.250	0.001	0.249
2022	0.274	0.000	0.249
2023	0.292	0.000	0.246

## Data Availability

Restrictions apply to the availability of these data. Data were obtained from INFARMED.

## References

[1] World Health Organization (2022). WHO Fungal Priority Pathogens List to Guide Research, Development and Public Health Action.

[2] Denning D.W. (2022). Antifungal drug resistance: An update. Eur. J. Hosp. Pharm..

[3] Shafiei M., Peyton L., Hashemzadeh M., Foroumadi A. (2020). History of the development of antifungal azoles: A review on structures, SAR, and mechanism of action. Bioorg. Chem..

[4] Fisher M.C., Alastruey-Izquierdo A., Berman J., Bicanic T., Bignell E.M., Bowyer P., Bromley M., Brüggemann R., Garber G., Cornely O.A. (2022). Tackling the emerging threat of antifungal resistance to human health. Nat. Rev. Microbiol..

[5] World Health Organization (2023). Web Annex A. World Health Organization Model List of Essential Medicines–23rd List, 2023.

[6] Pathadka S., Yan V.K.C., Neoh C.F., Al-Badriyeh D., Kong D.C.M., Slavin M.A., Cowling B.J., Hung I.F.N., Wong I.C.K., Chan E.W. (2022). Global Consumption Trend of Antifungal Agents in Humans From 2008 to 2018: Data From 65 Middle- and High-Income Countries. Drugs.

[7] Adriaenssens N., Coenen S., Versporten A., Goossens H. (2013). Outpatient systemic antimycotic and antifungal use in Europe: New outcome measure provides new insight. Int. J. Antimicrob. Agents.

[8] Azevedo M.M.S., Cruz L., Pina-Vaz C., Gonçalves-Rodrigues A. (2016). An overview about the medical use of antifungals in Portugal in the last years. J. Public Health Policy.

[9] Nunes A.M., Ferreira D.F.d.C. (2023). Evaluating Portuguese Public Hospitals Performance: Any Difference before and during COVID-19?. Sustainability.

[10] Tribunal de Contas (2020). COVID-19-Impacto na Atividade e no Acesso ao SNS. Relatório nº 5/2020-OAC 2ª Secção.

[11] Vieira-Baptista P., Stockdale C.K., Sobel J. (2023). International Society for the Study of Vulvovaginal Disease Recommendations for the Diagnosis and Treatment of Vaginitis.

[12] de Noronha N., Moniz M., Gama A., Laires P.A., Goes A.R., Pedro A.R., Dias S., Soares P., Nunes C. (2022). Non-adherence to COVID-19 lockdown: Who are they? A cross-sectional study in Portugal. Public Health.

[13] Azevedo M.J., Araujo R., Campos J., Campos C., Ferreira A.F., Falcão-Pires I., Ramalho C., Zaura E., Pinto E., Sampaio-Maia B. (2023). Vertical Transmission and Antifungal Susceptibility Profile of Yeast Isolates from the Oral Cavity, Gut, and Breastmilk of Mother-Child Pairs in Early Life. Int. J. Mol. Sci..

[14] Rolo J., Faria-Gonçalves P., Barata T., Oliveira A.S., Gaspar C., Ferreira S.S., Palmeira-de-Oliveira R., Martinez-de-Oliveira J., Costa-de-Oliveira S., Palmeira-de-Oliveira A. (2021). Species Distribution and Antifungal Susceptibility Profiles of Isolates from Women with Nonrecurrent and Recurrent Vulvovaginal Candidiasis. Microb. Drug Resist..

[15] Simões-Silva L., Silva S., Santos-Araujo C., Sousa J., Pestana M., Araujo R., Soares-Silva I., Sampaio-Maia B. (2017). Oral Yeast Colonization and Fungal Infections in Peritoneal Dialysis Patients: A Pilot Study. Can. J. Infect. Dis. Med. Microbiol..

[16] Sabino R., Gonçalves P., Martins Melo A., Simões D., Oliveira M., Francisco M., Viegas C., Carvalho D., Martins C., Ferreira T. (2021). Trends on Aspergillus Epidemiology-Perspectives from a National Reference Laboratory Surveillance Program. J. Fungi.

[17] Fernandes M.Z., Caetano C.F., Gaspar C., Oliveira A.S., Palmeira-de-Oliveira R., Martinez-de-Oliveira J., Rolo J., Palmeira-de-Oliveira A. (2023). Uncovering the Yeast Diversity in the Female Genital Tract: An Exploration of Spatial Distribution and Antifungal Resistance. Pathogens.

[18] Monteiro C., Pinheiro D., Maia M., Faria M.A., Lameiras C., Pinto E. (2019). Aspergillus species collected from environmental air samples in Portugal-molecular identification, antifungal susceptibility and sequencing of cyp51A gene on A. fumigatus sensu stricto itraconazole resistant. J. Appl. Microbiol..

[19] Amorim A., Guedes-Vaz L., Araujo R. (2010). Susceptibility to five antifungals of Aspergillus fumigatus strains isolated from chronically colonised cystic fibrosis patients receiving azole therapy. Int. J. Antimicrob. Agents.

[20] Costa-de-Oliveira S., Pina-Vaz C., Mendonça D., Gonçalves Rodrigues A. (2008). A first Portuguese epidemiological survey of fungaemia in a university hospital. Eur. J. Clin. Microbiol. Infect. Dis..

[21] Sabino R., Veríssimo C., Brandão J., Alves C., Parada H., Rosado L., Paixão E., Videira Z., Tendeiro T., Sampaio P. (2010). Epidemiology of candidemia in oncology patients: A 6-year survey in a Portuguese central hospital. Med. Mycol..

[22] Faria-Ramos I., Neves-Maia J., Ricardo E., Santos-Antunes J., Silva A.T., Costa-de-Oliveira S., Cantón E., Rodrigues A.G., Pina-Vaz C. (2014). Species distribution and in vitro antifungal susceptibility profiles of yeast isolates from invasive infections during a Portuguese multicenter survey. Eur. J. Clin. Microbiol. Infect. Dis..

[23] Fernandes Â., Azevedo N., Valente A., Dias M., Gomes A., Nogueira-Silva C., Henriques M., Silva S., Gonçalves B. (2022). Vulvovaginal candidiasis and asymptomatic vaginal colonization in Portugal: Epidemiology, risk factors and antifungal pattern. Med. Mycol..

[24] Pinto E., Ribeiro I.C., Ferreira N.J., Fortes C.E., Fonseca P.A., Figueiral M.H. (2008). Correlation between enzyme production, germ tube formation and susceptibility to fluconazole in Candida species isolated from patients with denture-related stomatitis and control individuals. J. Oral Pathol. Med..

[25] Carvalhinho S., Costa A.M., Coelho A.C., Martins E., Sampaio A. (2012). Susceptibilities of Candida albicans mouth isolates to antifungal agents, essentials oils and mouth rinses. Mycopathologia.

[26] Cavaleiro I., Proença L., Félix S., Salema-Oom M. (2013). Prevalence of yeast other than Candida albicans in denture wearers. J. Prosthodont..

[27] Figueiral M.H., Fonseca P., Lopes M.M., Pinto E., Pereira-Leite T., Sampaio-Maia B. (2015). Effect of Denture-Related Stomatitis Fluconazole Treatment on Oral Candida albicans Susceptibility Profile and Genotypic Variability. Open Dent. J..

[28] Silva A.P., Miranda I.M., Lisboa C., Pina-Vaz C., Rodrigues A.G. (2009). Prevalence, distribution, and antifungal susceptibility profiles of Candida parapsilosis, C. orthopsilosis, and C. metapsilosis in a tertiary care hospital. J. Clin. Microbiol..

[29] Bienvenu A.L., Bestion A., Pradat P., Richard J.C., Argaud L., Guichon C., Roux S., Piriou V., Paillet C., Leboucher G. (2022). Impact of COVID-19 pandemic on antifungal consumption: A multicenter retrospective analysis. Crit. Care.

[30] Mulet Bayona J.V., Tormo Palop N., Salvador García C., Fuster Escrivá B., Chanzá Aviñó M., Ortega García P., Gimeno Cardona C. (2021). Impact of the SARS-CoV-2 Pandemic in Candidaemia, Invasive Aspergillosis and Antifungal Consumption in a Tertiary Hospital. J. Fungi.

[31] Alhumaid S., Al Mutair A., Al Alawi Z., Alshawi A.M., Alomran S.A., Almuhanna M.S., Almuslim A.A., Bu Shafia A.H., Alotaibi A.M., Ahmed G.Y. (2021). Coinfections with Bacteria, Fungi, and Respiratory Viruses in Patients with SARS-CoV-2: A Systematic Review and Meta-Analysis. Pathogens.

[32] Despacho n.º 1150/2021, de 28 de Janeiro. https://diariodarepublica.pt/dr/detalhe/despacho/1150-2021-155575942.

[33] Statistics Portugal. https://www.ine.pt/xportal/xmain?xpgid=ine_main&xpid=INE.

[34] WHO Collaborating Centre for Drug Statistics Methodology ATC/DDD Index 2024. https://atcddd.fhi.no/atc_ddd_index/.

